# How to Use Heart Rate Variability: Quantification of Vagal Activity in Toddlers and Adults in Long-Term ECG

**DOI:** 10.3390/s20205959

**Published:** 2020-10-21

**Authors:** Helmut Karl Lackner, Marina Tanja Waltraud Eglmaier, Sigrid Hackl-Wimmer, Manuela Paechter, Christian Rominger, Lars Eichen, Karoline Rettenbacher, Catherine Walter-Laager, Ilona Papousek

**Affiliations:** 1 Division of Physiology, Otto Loewi Research Center, Medical University of Graz, 8010 Graz, Austria; marina.eglmaier@uni-graz.at (M.T.W.E.); christian.rominger@uni-graz.at (C.R.); 2 Institute of Medical Engineering, Graz University of Technology, 8010 Graz, Austria; 3 Department of Psychology, Educational Psychology Unit, University of Graz, 8010 Graz, Austria; sigrid.hackl-wimmer@uni-graz.at (S.H.-W.); manuela.paechter@uni-graz.at (M.P.); 4 Department of Early Childhood Education, University of Graz, 8010 Graz, Austria; lars.eichen@uni-graz.at (L.E.); karoline.rettenbacher@uni-graz.at (K.R.); catherine.walter-laager@uni-graz.at (C.W.-L.); 5 Department of Psychology, Biological Psychology Unit, University of Graz, 8010 Graz, Austria

**Keywords:** dynamic adaptation, autonomic nervous system, short-term variability, wearable biomedical sensing, methodological considerations, signal preprocessing, artifact detection, ECG derived respiration, Poincaré plot

## Abstract

Recent developments in noninvasive electrocardiogram (ECG) monitoring with small, wearable sensors open the opportunity to record high-quality ECG over many hours in an easy and non-burdening way. However, while their recording has been tremendously simplified, the interpretation of heart rate variability (HRV) data is a more delicate matter. The aim of this paper is to supply detailed methodological discussion and new data material in order to provide a helpful notice of HRV monitoring issues depending on recording conditions and study populations. Special consideration is given to the monitoring over long periods, across periods with different levels of activity, and in adults versus children. Specifically, the paper aims at making users aware of neglected methodological limitations and at providing substantiated recommendations for the selection of appropriate HRV variables and their interpretation. To this end, 30-h HRV data of 48 healthy adults (18–40 years) and 47 healthy toddlers (16–37 months) were analyzed in detail. Time-domain, frequency-domain, and nonlinear HRV variables were calculated after strict signal preprocessing, using six different high-frequency band definitions including frequency bands dynamically adjusted for the individual respiration rate. The major conclusion of the in-depth analyses is that for most applications that implicate long-term monitoring across varying circumstances and activity levels in healthy individuals, the time-domain variables are adequate to gain an impression of an individual’s HRV and, thus, the dynamic adaptation of an organism’s behavior in response to the ever-changing demands of daily life. The sound selection and interpretation of frequency-domain variables requires considerably more consideration of physiological and mathematical principles. For those who prefer using frequency-domain variables, the paper provides detailed guidance and recommendations for the definition of appropriate frequency bands in compliance with their specific recording conditions and study populations.

## 1. Introduction

Recent advances in technical development such as wearable biomedical sensing through smart clothing and smart mobile devices make physiological measurements of high technical quality including recordings of the electrocardiogram (ECG) available to scientific and medical staff and non-professionals alike [1,2]. Short-term ECG measurements and the segmental analysis of long-term ECG recordings provide information about two distinct but overlapping processes: (1) the complex and dynamic relationship between the sympathetic and parasympathetic branches of the autonomic nervous system and (2) regulatory mechanisms that control the heart rate [3]. These regulatory mechanisms include respiratory-driven acceleration and deceleration of the heart rate (respiratory sinus arrhythmia, RSA) via the nervus vagus, the baroreceptor reflex for short-term blood pressure regulation, and rhythmic changes in the vascular tone. In addition, a variety of excellent non-commercial and commercial software are available, which provide the quantification of inter-beat intervals (R–R intervals) and a—for the most part automatic—analysis of heart rate and heart rate variability (HRV) patterns in short- and long-term recordings [4,5,6,7]. In other words, using smart mobile devices, HRV provides a seemingly simple opportunity to examine the interaction between sympathetic and parasympathetic nervous system activities non-invasively, which may supply with useful information about a number of physiological situations. For that reason, these developments also engage great interest of professionals in non-medical fields. However, the correct interpretation of such recordings needs some physiological and mathematical background to avoid misinterpretations. However, also experienced users and researchers may not always be fully aware of all fundamental principles and weaknesses of the measures they use and thus may not be immune from falling into an interpretation pitfall from time to time. The present paper aims at providing some support in that regard and presenting detailed methodological clarification as well as new data material for illustration.

In the literature, reports of numerous studies using heart rate variability can be found that aimed to detect the physiological effects of conditions involving the experience of stress, emotional stimulation, or various kinds of physical activity [8,9,10]. The majority of these studies were done in a laboratory environment in healthy adults or adult patients with medical conditions. However, HRV studies are also available for other groups such as neonates [11,12] and children of school-age [13,14,15,16]. Most of these studies only assessed short-term HRV, using recording periods of one to several minutes during experimental manipulations [14,15,17,18,19].

By means of state-of-the art devices, long-term ECG recording and the subsequent HRV analysis can be easily accomplished. The possibility of using ECG data of 24-h recordings not only for clinical evaluation of cardiac function but also, for instance, to gain information on varying levels of stress, physical activity, and so forth is tempting. To analyze and interpret this amount of data, at least semi-automatic algorithms are necessary. For this reason, we think it is necessary to take a step back and discuss basic requirements. While well-recognized guidelines on the recording and computing of HRV variables in terms of signal quality, minimum length of epochs, etc. are available [20,21], only little information is available on the extraction of HRV variables from longer-term ECG recordings in real-life conditions. In that context, also the identification of technical and physiological artifacts following QRS complex detection is of particular importance [22].

The time-domain measures of HRV, of which SDNN and RMSSD are the most common ones, are easy to calculate: SDNN refers to the standard deviation of all regular R-R intervals; RMSSD refers to the square root of the mean squared differences of successive R-R intervals. However, important points to consider are (1) that there is no time information in the SDNN [20] and (2) the high-pass filter characteristic of RMSSD [23], which might in addition depend on the heart rate (R-R interval).

Since the seminal work of Akselrod and co-workers in 1981 [24], the power spectrum analysis of heart rate fluctuation—that is, the calculation of frequency-domain variables of HRV—is a widely accepted and used method. The low-frequency (LF) band power in the frequency range of 0.04–0.15 Hz, considered to represent sympathetic and parasympathetic activity, and the high-frequency (HF) band power in the range of 0.15–0.40 Hz, considered to represent parasympathetic activity and the influence of respiration [20], are of widespread use. However, particularly the definition of the HF band is a subject of discussion. For instance, a respiration rate less than 9 min^−1^ (0.15 Hz) can affect the LF band power, and a respiration rate higher than 24 min^−1^ (0.40 Hz) will not be completely included in the HF component of the power spectrum. Therefore, a wider frequency band and other frequency limits for the HF band were proposed for infants due to the different physiological range of their respiration rate [25]. For instance, similar problems were indicated in adults during exercising [25]. Moreover, the physiological interpretation of the common HF and LF bands is not fully clarified. For example, the sympathetic nervous system does not appear to produce rhythms above 0.1 Hz [3], while the parasympathetic system affects heart rate rhythms down to 0.05 Hz. It is also a continuing matter of top-level debate whether the RSA has its origin in central mechanisms or in the baroreflex mechanism [26,27].

In addition to the time-and frequency-domain methods, a variety of other methods of HRV calculation were introduced, such as nonlinear methods. The most commonly used nonlinear method is based on the Poincaré plot, which is a scatter plot of successive R-R intervals that produces two standard deviation measures. SD1, the standard deviation of the projection of the Poincaré plot on the line normal to the line of identity describes the short-term variability. SD2, the projection on the line of identity, describes the long-term variability [28]. It is important to note that the mathematical connection between SD1 and RMSSD is (SD1 = RMSSD/sqrt(2)): that is, these variables contain redundant information in a statistical sense [3,29].

Taken together, the recording of HRV is an easy to use non-invasive method that can provide useful information about physiological functioning in a number of relevant situations. Nonetheless, even when using the most common variables, pitfalls in the interpretation may occur. The purpose of the present paper is to provide a helpful notice of HRV monitoring issues, with special emphasis on the monitoring over long periods, across periods with different activity levels, and in adults versus children. To this end, the paper supplies a detailed methodological discussion as well as new data material. Long-term electrocardiographic recordings made in real-life conditions, which additionally included several levels of activity and, therefore, wide ranges of heart rate and respiration rate, are analyzed in detail and with particular methodological rigor. Since many important issues seem to be largely neglected as regards the determination of the widely used HF band power, this is the main focus of the paper. We evaluate the adequacy of frequency-domain as well as time-domain HRV variables for data gained in recording conditions with different activity levels and study populations. As various recommendations for the selection of frequency ranges representing the high-frequency (HF) component of heart rate variability exist in the literature, the HF component is calculated for several frequency bands. Additionally, the nonlinear HRV variable SD1 is used, which is an adequate measure of short-term variability [3] and equivalent to the standard time-domain HRV metric RMSSD (root mean square successive differences of R-R intervals). In addition to demonstrating the HRV variables’ fundamental properties, the accordance between the band power derived from the several HF band definitions and SD1 is the main focus of the analysis. This helps to determine the adequacy of the HF frequency bands considering different recording conditions and age groups. Additional analyses inform about the gain in captured high-frequency variability by using a broader frequency band compared to narrower bands, and if and in which way that depends on recording conditions and age. The multiplex information is finally integrated with the objectives of demonstrating the impact of neglected methodological issues and of providing substantiated recommendations for the selection of appropriate HRV variables and their interpretation. As there is a lack of research on (and lack of awareness, for that matter) the appropriateness of common HF band definitions in young children, particular attention is given to this age group.

## 2. Materials and Methods

### 2.1. Participants

Forty-nine healthy adults (22.8 ± 4.4 years, range: 18–40 years) and fifty-one healthy toddlers (27.8 ± 5.0 months, range: 16–37 months), all of Caucasian ethnicity, participated in the study including long-term ECG monitoring. Exclusion criteria were prescription medication, and in addition, in toddlers, premature birth (i.e., less than 37 weeks of gestation) or diagnosis of developmental disorder.

ECG monitoring started at Monday to Thursday at around 7:30–9:00 a.m. with a scheduled duration of thirty to thirty-three hours. In addition, participants (in case of toddlers’ parents and kindergarten teachers) were asked to keep a simple activity log to record sleep during the night and day, mealtimes, and dominant activities during the day with begin and end times. The plausibility of the activity records was checked by the data of the acceleration sensor of the used device in terms of body position and dynamics of the signal (see below).

Informed consent was obtained from the participants/parents of the toddlers, after they received a detailed explanation of the study. The study was performed in accordance with the 1964 Declaration of Helsinki in its current form and was approved by the authorized ethics committee (39/52/63 ex 2017/18; 28 May 2018).

### 2.2. Data Acquisition and Preprocessing

#### 2.2.1. Data Acquisition

Continuous ECG monitoring was carried out with a portable device (eMotion Faros 180^TM^, Bittium Biosignals Ltd., Kuopio, Finland, single-channel ECG, Einthoven Lead II set-up, sampling rate 1 kHz; weight: 13 g). The device included a 3D acceleration sensor. The raw data were converted from EDF to MATLAB^®^ data format for further analysis.

#### 2.2.2. Artifact Handling

To check the quality of the physiological data and calculate the inter-beat interval time series, we have been consistently using for several years a semi-automatic artifact-handling device developed by our research group. In brief, its main criteria are:

1. The pattern of the QRS complex and the time of occurrence within the ECG to identify “ectopic beats”
2. Physiological limits on an individual, age-depending basis, and
3. The maximal percentage of change in relation to the standard deviation of the signal.

As regards points 2 and 3 above, our algorithm was inspired by Cheung [30] and Berntson et al. [31], utilizing the additional advantage of incorporating the whole information of the ECG and not the R-R intervals (RRI) alone. The physiological limits and percentage of change are calculated on an individual basis based on the envelope of the R-R intervals using the Hilbert transformation. To obtain an R-R interval time series with equidistant time steps for the calculation of frequency-domain HRV variables, the beat-to-beat values were resampled at 4 Hz, using piecewise cubic spline interpolation after the described semi-automatic artifact correction. In the process, invalid data were marked for the determination of the percentage of valid data (see below).

#### 2.2.3. Calculation Criteria

Further requirements for the calculation of the HRV variables such as the “quasi-stationarity” of the inter-beat intervals and the appropriate estimation of the frequency-domain variables were checked by fundamental mathematical equations (e.g., Parseval theorem) in additional steps during the calculation of the HRV variables (criteria see below).

Due to the high dynamic of the heart rate (or RRI, respectively) within daily activity, especially in toddlers, short 180 s segments were used for the quantification of HRV variables, with a step width of 30 s (see Section 2.3). The 180 s interval was chosen in order to ensure the stability and stationarity of the signal, taking into account that the segment should be no less than approximately ten times the wavelength of the lower frequency bound of the investigated component. As we focused on the short-term variability, 0.15 Hz (9 s) was the limit. However, 180 s enabled us to estimate the long-term variability, too.

In addition, the respiration rate was calculated using a modified EDR (ECG-derived respiration) algorithm based on the description of Moody and colleagues in 1985 [32], with a modified pattern recognition after data normalization and with individual physiological ranges for respiration based on the age of the participant and the reported activity, and the percentage of valid respiration cycles in the 180 s segments. However, it is essential to state already at this point that the calculation of the respiration rate in a high dynamic heart rate time series is difficult and must take the physiologically plausible range of the respiration rate into account (which is different in children and adults).

Only 180 s segments that fulfilled the following criteria were used for further analysis:

1. 95% of the heart beats recalculated on a time basis had to be valid. To be more precise, 9 s of replaced, interpolated data were allowed in total, whereby only 3.6 s (2%) of interpolated data were allowed in a row.
2. For the confirmation of “quasi-stationarity”, a simple but very efficient procedure was used. In brief, the ratio of the standard deviations of the detrended time series with (1) removed mean value (STD_0_) and (2) removed second order trend (STD_2_) was calculated; and only ratios STD_2_/STD_0_ between 0.8 and 1.1 were considered as valid.
3. The Parseval theorem had to be fulfilled with an accuracy of at least 95% for the power spectral density (PSD) estimate in the range of 0–1.04 Hz and (STD_2_)^2^; that is, the variance of the resampled, second-order detrended R-R time series. That is, only segments with (almost) equivalent information content were considered valid and used for further analysis.
4. Finally, the calculation of the respiratory rate must have been possible in at least one-third of the respective 180 s segment.

Furthermore, based on the reported activity logs, the time period between 12:00 p.m. and 2:00 p.m. was excluded from the analysis, because toddlers usually took an afternoon nap after lunch, which was validated by the data from the acceleration sensor. In addition, occasionally reported sleep periods at other times of the day were excluded from the analysis as well. Due to the strict exclusion criteria, the lower limit of valid data that had to be available from a participant in order to being entered in the data analysis was set to 30%; but at least the data of 4 h of the total time during the day and during sleeping hours must have been acceptable, respectively.

#### 2.2.4. Compilation of Final Data Set

Finally, the data of 48 adults (22.7 ± 4.5 years, range: 18–40 years) and 47 toddlers (27.7 ± 5.0 months, range: 16–37 months) were used for further analysis. In detail, in adults, the rate of valid data was 56.0 ± 9.5% (range: 33.2–79.6%) during the day and 80.7 ± 11.1% (45.6–93.5%) during sleep. In toddlers, 52.5 ± 11.4% (range: 33.1–73.9%) during the day and 85.9 ± 10.5% (37.4–96.3%) during sleep were acceptable for further analysis (*time of day*: *F*(1,93) = 422.9, *p* < 0.001; *time of day × age group*: *F*(1,93) = 9.7, *p* = 0.002). Clearly, more data would have entered the analysis, if only the criteria of point 1 would have been applied, which only considers the normality of heart beats and technical artifacts and is the most commonly used procedure of data preprocessing in the field: In that case, the average rate of valid data would have been 91.5 ± 5.90% (range: 74.3–99.6%) in adults during the day and 91.5 ± 11.4% (51.2–100%) in adults during sleep. The rates for the toddlers would have been 80.1 ± 15.1% (range: 50.6–100%) during the day and 93.3 ± 10.2% (45.7–100%) during sleep (*time of day*: *F*(1,93) = 18.2, *p* < 0.001; *time of day × age group*: *F*(1,93) = 17.8, *p* < 0.001).

In addition to the separate calculation of the variables for sleeping times and during the day, a separate analysis was done with the data during the day recorded during periods of high versus low levels of activity. These periods were selected on the basis of (a) the heart rate, (b) the dynamic of the acceleration data, and (c) the reported activities as plausibility criterion, with a lower limit of at least 90 min of available data.

On average, the ECG monitoring time for the final sample was 29.9 ± 1.9 h (range: 25.5–33.0 h) in adults and 29.6 ± 0.8 h (range: 26.3–30.0 h) in toddlers, *F*(1,93) = 1.0, *p* = 0.32. The reported sleep duration during the night was 8.1 ± 1.2 h (range: 5.0–10.3 h) in adults and 10.7 ± 0.9 h (range: 9.0–13.0 h) in toddlers, *F*(1,93) = 138.8, *p* < 0.001.

### 2.3. Analysis Procedure

As already mentioned, 180 s segments were used for the calculation of the HRV variables, with a step width of 30 s. Mean heart rates (bpm) and the medians of the respiration rate (min^−1^) were additionally calculated.

#### 2.3.1. Time Domain HRV Variables

SDNN, the standard deviation of the regular R-R intervals and RMSSD, the square root of the mean squared differences of successive R-R intervals were calculated.

#### 2.3.2. Nonlinear HRV Variables

Nonlinear parameters based on the Poincaré plot interpretation are expressed in the standard deviation of the short-term R-R interval variability, SD1 (ms), and the standard deviation of the long-term R-R interval variability, SD2 (ms). SD1 and SD2 were natural logarithm transformed. However, it has to be noted that SD1 and RMSSD (root mean square successive differences of R-R intervals) are identical heart rate variability metrics as shown, e.g., by Brennan et al. [28] and Ciccone et al. [29]; and SD2 and SDNN are very highly correlated. Therefore, while the time-domain variables RMSSD and SDNN were calculated by default, RMSSD data are not reported in this manuscript to prevent statistical redundancy.

#### 2.3.3. Frequency Domain HRV Variables

**Figure 1:**
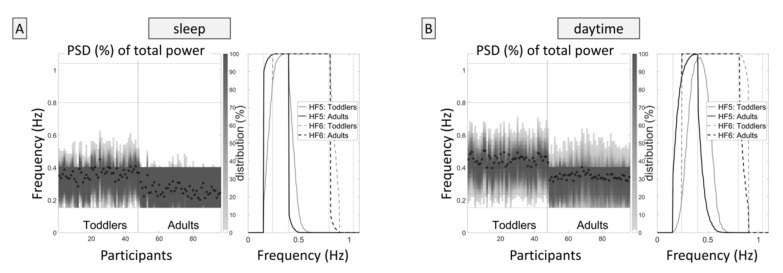
Dynamically adjusted frequency bands. The figure on the left side shows the distribution of the frequency ranges for each participant during sleep. (**A**), each column represents one participant. The shade of the columns represents the percentage of the power spectral density (PSD) in the respective frequency (percentage of the respective participant’s total power). The dots represent the respiration frequency of each participant (the arithmetic mean of all dots for toddlers equals the value given in Table 1, i.e., 0.36 Hz or 21.7 min^−1^). (**B**) shows the percentages of participants in whom variability in the respective frequencies was continuously present, that is, in all 180 s segments over the entire recording period, displayed for HF5 and HF6. The figure illustrates how well (or poorly) the frequency bands captured the actual heart rate variability in adults and toddlers: In the majority of adults, but not in toddlers, the dominant frequencies are in the recommended frequency range for short-term heart rate variability (HRV, 0.15–0.40 Hz). The right figure shows the dynamic frequency band adaptation for the day.

Power spectral density (PSD) estimates were calculated for the 180 s segments via Burg’s method (model order 24) after removing the trend (2nd order). Given the two different age groups and the respective different recommendations in the literature, the high-frequency component (HF) of the heart rate variability was calculated for multiple frequency bands:

1. HF1: 0.15–0.40 Hz, the recommended frequency band for adults [20]
2. HF2: 0.15–0.80 Hz: frequency band proposed for children by Alkon et al. [33,34]
3. HF3: 0.24–1.04 Hz: further frequency band proposed for children [35,36,37]
4. HF4: 0.15–1.04 Hz: the combination of the proposed frequency bands for children in terms of the maximal range
5. Dynamically adjusted frequency bands based on the median respiration rate within the 180 s segment with:
  (a) HF5: a minimum frequency of 0.15 Hz and a range of 0.25 Hz, equal to the band width of HF1 (see Figure 1, please). The optimal frequency range was calculated based on the overlapping frequency range of HF1 and HF3, that is, the range from 0.24–0.40 Hz (center 0.32 Hz), and the difference of the current respiration frequency (in the same 180 s segment) from this center frequency of 0.32 Hz. Consequently, a respiration frequency of 0.32 Hz (respiration rate of 19.2 min^−1^) or lower resulted in a frequency band ranging from 0.15 to 0.40 Hz; a respiration rate of 24 min^−1^ (0.40 Hz) resulted in a frequency band ranging from 0.23 to 0.48 Hz; a respiration rate of 30 min^−1^ (0.50 Hz) resulted in a frequency band ranging from 0.33 to 0.58 Hz; and so forth.
  (b) HF6: same as HF5 but with a range of 0.65 Hz, equal to the band width of HF2 (limits: minimum frequency 0.15 Hz, maximum frequency 0.93 Hz).

Due to skewed distributions, all HF variables were natural logarithm transformed.

#### 2.3.4. Prevalent Frequency Ranges

**Figure 2:**
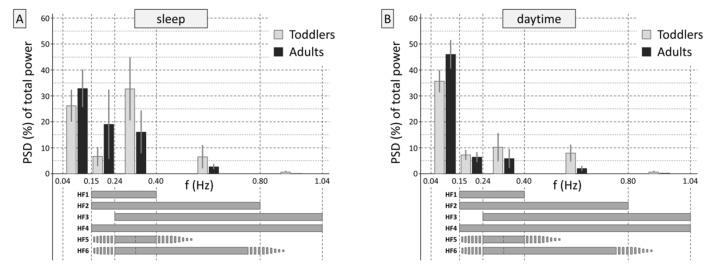

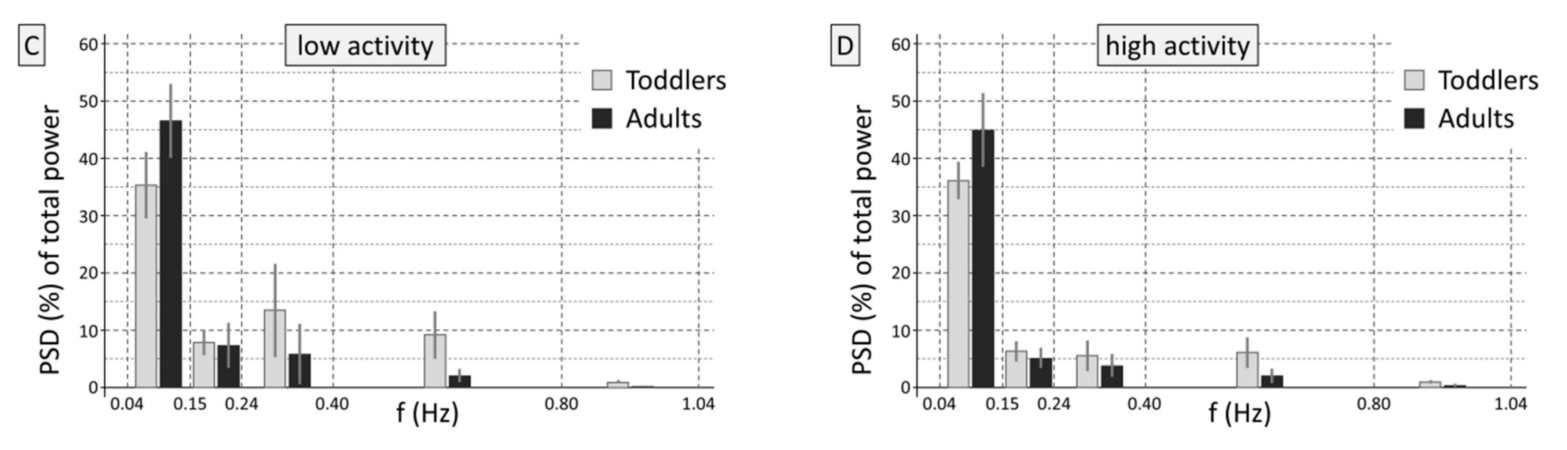
Prevalent frequency ranges (frequency domain). (**A**) shows the proportions of normalized high-frequency (HF) power in the frequency ranges of interest, which were averaged across participants and expressed as percentages of the total heart rate (HR) variability. Adding up the depicted values to HF1 (0.15–0.40 Hz, i.e., 2nd + 3rd column, see schematic bars below parts (**A**,**B**), HF2 (0.15–0.80 Hz), HF3 (0.24–1.04 Hz), and HF4 (0.15–1.04 Hz) provides an impression of the power spectral density estimates for these frequency bands, independently from the RRI level. Furthermore, the proportion of long-term variability can be seen in the low-frequency range (0.04–0.15 Hz). During sleep, the difference in the dominant HF frequency band between toddlers and adults—in toddlers 0.24–0.40 Hz—can be seen clearly. (**B**) shows the normalized HF power during the day, and (**C**,**D**) displays the separate analyses for periods with low and high levels of activity during the day.

The percentage of PSD in the frequency bands 0.15–0.24 Hz, 0.24–0.40 Hz, 0.40–0.80 Hz, and 0.80–1.04 Hz of the total frequency spectrum from 1/180 to 1.04 Hz were calculated to show the dominant frequency parts (see Figure 2, please). The two groups showed different frequency distributions during sleep (interaction *frequency band × age group*, *F*(4,372) = 46.9, *p* < 0.001) and during the day (interaction *frequency band × age group*, *F*(4,372) = 44.8, *p* < 0.001). The analysis for low and high activity levels additionally corroborated these results (*F*(4,372) = 54.8, *p* < 0.001 and *F*(4,372) = 75.3, *p* < 0.001, respectively).

**Figure 3:**
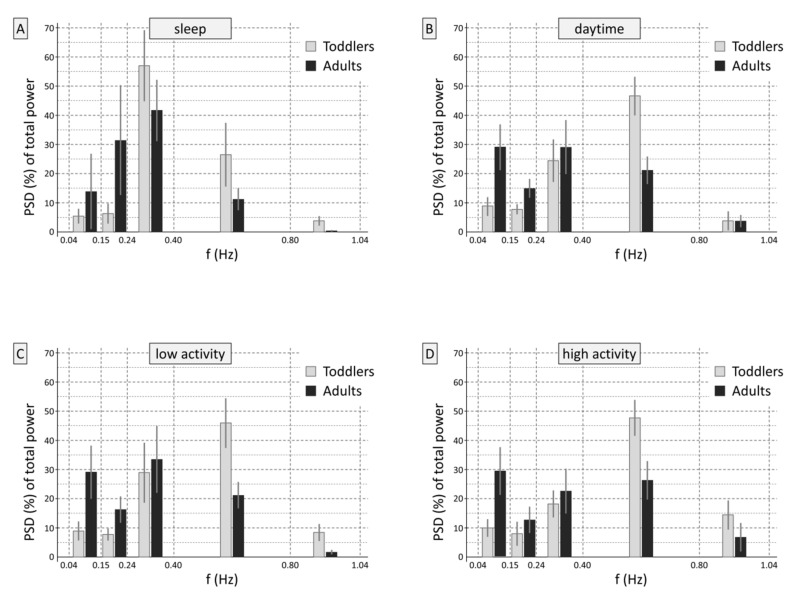
Prevalent frequency ranges in root mean square successive differences of R-R intervals (RMSSD)/SD1. (**A**) shows the proportions of normalized power for the time series of differences of successive R-R intervals in the frequency ranges of interest, which were averaged across participants and expressed as percentages of the total HR variability. Adding up the depicted values to HF1 (0.15–0.40 Hz), HF2 (0.15–0.80 Hz), HF3 (0.24–1.04 Hz), and HF4 (0.15–1.04 Hz) provides an impression of the power spectral density estimates for these frequency bands, independently from the RRI level. Furthermore, the proportion of long-term variability can be seen in the low-frequency range (0.04–0.15 Hz). During sleep, the difference in the dominant HF frequency band between toddlers and adults—in toddlers, 0.24–0.40 Hz—can be seen clearly. (**B**) shows the normalized HF power during the day, and (**C**,**D**) displays the separate analyses for periods with low and high levels of activity during the day. Please compare Figure 2 and Figure 3 to gain an impression of the high-pass filter effect of the calculation of RMSSD or SD1.

However, as stated in the introduction, RMSSD and due to the mathematical connection SD1 as well, entail high-pass filter characteristics. To demonstrate this, the signal preprocessing was repeated by resampling the time series of differences of successive R-R intervals (RRI_n+1_-RRI_n_) at 4 Hz, again using piecewise cubic spline interpolation. Power spectral density (PSD) estimates were calculated for the 180 s segments using Burg’s method (model order 24) without trend correction, and the percentages of PSD in the frequency bands in the ranges of 0.15–0.24 Hz, 0.24–0.40 Hz, 0.40–0.80 Hz, and 0.80–1.04 Hz of the total frequency spectrum from 1/180 to 1.04 Hz were calculated. Figure 3 shows that the two groups showed different frequency distributions in the time series of successive RRI, on which SD1 is based (sleep: interaction *frequency band × group*, *F*(4,372) = 58.7, *p* < 0.001; during the day: *F*(4,372) = 182.6, *p* < 0.001, for low and high activity levels: *F*(4,372) = 141.9, *p* < 0.001 and *F*(4,372) = 147.5, *p* < 0.001, respectively.

Please compare Figure 2 and Figure 3 for similarities and differences. Figure 3 shows in which frequencies the heart rate variability expressed as RMSSD or SD1 is predominantly rooted. Differences are evident compared to the variables in the frequency domain (Figure 2). The figure impressively illustrates the high-pass filter effect of computing RMSSD/SD1: When using the time-based RMSSD or SD1, the frequency shares of interest come to the fore by the method of computation alone, without the necessity to define specific boundaries.

#### 2.3.5. Degree of Short-Term Variability Captured by a Specific Frequency-Domain HF Band Compared to SD1

In order to estimate the degree of mathematical connection between each of the six specific HF bands (HF1 to HF6) and SD1, Pearson correlation coefficients r_(ln(SD1) * ln(HF1–6))_ between SD1 and the absolute band power in the six HF bands were calculated for each participant. Each correlation was calculated using the respective HF band power values (y) and the SD1 values (x) of all valid 180 s segments of a participant as the bivariate dataset. This resulted in 6 (HF1, HF2, HF3, HF4, HF5, HF6) × 4 (sleep, total daytime, low-activity daytime, high-activity daytime) = 24 correlation coefficients per participant. To ensure appropriate data quality (scale of measurement) for subsequent parametric statistical analyses (see Section 2.4), the Pearson correlation coefficients r_(ln(SD1) * ln(HF1–6))_ were Fisher’s Z’-transformed by Z’ _(ln(SD1) * ln(HF1–6))_ = 0.5*ln((1 + r_(ln(SD1) * ln(HF1–6))_)/(1 − r_(ln(SD1) * ln(HF1–6))_)).

### 2.4. Statistical Analysis of Accordance between Short-Term Variability Captured by the HF Bands and SD1

With the following inferential statistical analysis, it was evaluated whether the accordance between a frequency-domain based heart rate variability estimate and SD1 (expressed in the Z’-transformed correlation coefficients) differs depending on the tested age group (toddlers vs. adults) and the time of day/activity level (sleep vs. daytime, low activity vs. high activity periods in the daytime), and if these relationships are different for the frequency bands (HF1 to HF6). The final aim of the analysis was to reach a conclusion regarding which recording condition of the six frequency bands (HF1 to HF6) shows the highest accordance to SD1 in toddlers and which shows the highest accordance to SD1 in adults.

To this end, a 6 × 2 × 2 three-way analysis of variance was done with *frequency band* (HF1 to HF6, see Section 2.3.2) and *time of day* (sleep, daytime) as within-subjects factors, and *age group* (toddlers, adults) as the between-subject factor. The relevant effects in this analysis are the two-way interaction effect *frequency band by age group* (indicating, if significant, that it depends on the age group which frequency band shows higher accordance with SD1) and the three-way interaction effect *frequency band by time of day by age group* (indicating that it depends on the time of day (sleep, daytime) at which the ECG data were recorded, which frequency band shows higher accordance with SD1 in a particular age group). A second, analogous three-way analysis of variance was done with *frequency band* (HF1 to HF6) and *activity level* during the day (low, high) as within-subjects factors and *age group* (toddlers, adults) as the between-subject factor. Following common conventions, the F-values for the effects in analyses of variance are given in the format F(df_between_, df_within_), where df_between_ denotes the degrees of freedom between groups or conditions, and df_within_ denotes the degrees of freedom within groups or conditions.

### 2.5. Statistical Analysis of Differences in Basic Cardiac and Respiration Characteristics and Levels of Absolute Band Power in HF1 to HF6

Differences in mean heart rate levels, mean levels of total (SDNN), short-term (SD1), and long-term (SD2) variability, and respiration frequency between toddlers and adults were statistically compared using a 2 × 2 two-way analysis of variance for each variable, with *time of day* (or *activity level* in the daytime) as the within-subjects factor and *age group* as the between-subject factor.

Two 6 × 2 × 2 three-way analyses of variance were used to compare the levels of absolute band power in the frequency domain-based HRV variables (frequency bands H1 to H6). *Frequency band* and *time of day* (or *activity level* in the daytime) were the within-subjects factors in these analyses, and *age group* was the between-subject factor. Again, the effects of interest were the two-way interaction effect *frequency band by age group* (indicating that differences in absolute band power between the predefined frequency bands were different in toddlers vs. adults) and the three-way interaction effect *frequency band by time of day by age group* (indicating that such differences between toddlers and adults depend on the time of the recording (sleep or daytime). This analysis essentially helps determine if broader frequency bands bring a gain in the captured high-frequency variability compared to the narrower bands, and in which way this depends on the age of the participants and the recording conditions.

## 3. Results

### 3.1. Accordance between Short-Term Variability Captured by the Specific HF Bands and SD1

The results of the inferential statistical analysis confirmed that the accordance between a particular short-term variability estimate and SD1 varies in a complex way depending on the age group of the participants and the circumstances in which the recordings were made. In the first analysis of variance (analysis 1: sleep vs. daytime), the relevant interaction effect *frequency band × age group (F*(5,465) = 80.3, *p* < 0.001) was significant, which was additionally qualified by the also significant three-way interaction *frequency band × time of day × age group F*(5,465) = 41.7, *p* < 0.001). That means that the frequency band that shows higher accordance with SD1 in a particular age group depends on the conditions of the recording (sleep vs. daytime). 

The descriptive data are given in Table 1. The most important specific effects embedded in this statistical interaction are as follows. During sleeping hours, the largest average correlation in toddlers appeared between SD1 and the band power in the HF4 band (0.15–1.04 Hz), mean Z’_(ln(SD1) ∗ ln(HF4))_ = 2.51, which corresponds to a mean Pearson’s correlation coefficient of r = 0.987. Participants’ Pearson correlation coefficients r_(ln(SD1) ∗ ln(HF4))_ in this age group and recording condition (toddlers, sleep) range from r = 0.927 to 0.998. From the mean correlation coefficient of r = 0.987, it follows that SD1 and the band power in the HF4 band share 97.42% of their variance (r^2^ = 0.9742; range: 85.93%–99.96%). Scores for HF2 (0.15–0.80 Hz) were similar (see Table 1). The lowest mean correlation in toddlers was observed in HF1 (0.15–0.40 Hz), which is the commonly recommended high-frequency band for adults (r = 0.968, r^2^ = 0.937, range: r = 0.748 to 0.994). Similar to toddlers, during sleeping hours, adults showed the highest correlations between the band power in an HF band and SD1 in the bands HF2 (0.15–0.80 Hz) and HF4 (0.15–1.04 Hz). However, the lowest correlation in adults was observed in HF3 (0.24–1.04 Hz), which is a frequency band that was proposed for children. Here, the single correlation coefficients showed high variability, ranging from r = 0.362 to 0.992 (equivalent to a range of shared variance between HF3 and SD1 of 13.10% to 98.41%).

In the daytime, a different picture emerged. Toddlers showed the highest accordance between the band power in an HF band and SD1 in the HF3 band (0.24–1.04 Hz), with single correlation coefficients ranging from r = 0.922 to 0.996 (HF4 band: r = 0.885 to 0.996). In toddlers, the by far least accordance during the day was shown again between SD1 and the band power in HF1, which is the recommended frequency band for adults (0.15–0.40 Hz), range of correlation coefficients r = 0.777 to 0.987. In adults, the broader frequency bands that were proposed for children (HF2, 0.15–0.80 Hz; HF3, 0.24–1.04 Hz) showed similar correlations as the commonly used HF1 band (0.15–0.40 Hz).

Analysis 2, which took the activity levels in the daytime into account, showed similar results (*frequency band × age group* (*F*(5,465) = 63.5, *p* < 0.001; *frequency band × time of day × age group F*(5,465) = 15.2, *p* < 0.001). Please find the descriptive data in Table 1, which show that the differences between low- and high-activity periods in part mirror, and thus corroborate, those between sleeping and daytime hours. Accordances are lower during high- than during low-activity periods, particularly in toddlers. HF1, the recommended frequency band for adults (0.15–0.40 Hz), appeared as the least adequate frequency band for toddlers, even more so in periods of high activity.

### 3.2. Differences in Basic Cardiac and Respiration Characteristics

Descriptive data of basic cardiac and respiratory characteristics in the two age groups are displayed in Table 2. It is most important to note that the average respiration frequency of toddlers during sleep was 21.7 min^−1^—that is, 0.36 Hz. While this would be in the recommended HF range of adults (0.15–0.40 Hz), the average respiration frequency during the day was 0.45 Hz and thus higher in toddlers than adults (0.35 Hz; *age group*: *F*(1,93) = 440.2, *p* < 0.001; *time of day × age group*: *F*(1,93) = 0.7, *p* = 0.39).

Toddlers showed the expected higher heart rates [38] and a smaller impact of the time of day on their heart rates compared to adults (analysis 1: *age group*: *F*(1,93) = 357.8, *p* < 0.001; *time of day × age group*: *F*(1,93) = 7.7, *p* = 0.007). The additional analysis for low vs. high activity levels during the day (analysis 2) yielded similar results (*age group*: *F*(1,93) = 162.5, *p* < 0.001; *activity level × age group*: *F*(1,93) = 42.5, *p* < 0.001). The other variables showed similar patterns (please see Table 2).

### 3.3. Differences in Levels of Absolute Band Power in HF1 to HF6

The results of the inferential statistical analysis confirmed that it depends on the age group of the participants as well as the circumstances in which the recordings were made if and how much high-frequency variability is additionally captured by broader compared to narrower frequency bands (analysis 1, including time of day: *frequency band × age group, F*(5,465) = 141.5, *p* < 0.001, *frequency band × time of day × age group, F*(5,465) = 15.5, *p* < 0.001; analysis 2, including activity levels: *frequency band × age group, F*(5,465) = 122.4, *p* < 0.001, *frequency band × time of day × age group, F*(5,465) = 22.0, *p* < 0.001).

Please see Table 3 for the descriptive data. Essentially, the observed patterns are in line with the prevalent frequency ranges illustrated in Figure 2. The most important specific effect is that during sleeping hours, HF1 (0.15–0.40 Hz), which is the commonly recommended high-frequency band for adults, does not adequately capture the high-frequency variability in toddlers, but some variability also seems to be missed with the sole use of HF3, which is a broad band starting at 0.24 Hz (0.24–1.04 Hz) that was recommended for children.

In adults, too, some gain is brought by the range from 0.40 to 0.80 Hz compared to the HF1 range alone (0.15–0.40 Hz; see difference between HF1 and HF2 in Table 3). The frequency range > 0.80 Hz appears insignificant in toddlers as well as in adults. In the daytime, band power is highest in the broadest band (HF4, 0.15–1.04 Hz) in toddlers as well as in adults, but little is gained compared to HF2 (0.15–0.80 Hz), again suggesting that frequencies > 0.80 Hz are negligible.

## 4. Discussion

The physiological basis of heart rate variability can be described as a closed-loop control system of cardiovascular oscillations driven by the central autonomic network, which consists of the interplay between the components of the autonomic nervous system, brainstem modulatory nuclei, and cortical function. To describe such a complex system with mathematical means in terms of signal processing is almost impossible. Nevertheless, a variety of variables capturing components of heart rate variability have been introduced, and they yielded important findings based on sophisticated study designs. At the same time, the easy accessibility and applicability of devices for the recording of electrocardiographic data along with easy-to-use software for the display of standard heart rate variability variables tempt users to neglect the complex foundations on which they are based, which may give rise to pitfalls in their interpretation.

The present paper seeks to make users aware of neglected physiological and methodological foundations and limitations and to provide transparent recommendations for the selection of heart rate variability variables for particular recording conditions and study populations that go beyond popular recommendations. Its novel contribution primarily originates from the in-depth analysis of empirical data gained in real-life conditions, which additionally included several levels of activity and, therefore, wider ranges of heart and respiration rates compared to typical laboratory studies. Moreover, the present study included a sample of young children. As the appropriateness of common variable definitions in young children is much under-researched, the detailed analysis of heart rate variability characteristics in young children is another important contribution of the present paper. To allow substantiated and reliable recommendations, the data analysis was carried out with particular methodological rigor. Finally, the authors put emphasis on making the paper valuable also for users of heart rate variability variables who are not top experts in their specific physiological and mathematical foundations. To this end, we first discuss the most important physiological principles and the most important issues in data preprocessing. Then, we explain the properties of the primary HRV variables after signal preprocessing, which is mandatory for a reasonable interpretation of the empirically determined accordance between heart rate variability in the frequency domain (in different frequency bands) and the non-linear variable SD1, which adequately represents cardiac short-term variability [3]. Finally, the multiplex information is integrated with the aim of reaching clear recommendations for the selection of appropriate HRV variables and their interpretation.

### 4.1. Notable Physiological Principles

In toddlers in the age range of our study population, the heart rate ranges approximately from 80 to 140 bpm (while awake and resting); and the respiration rate ranges from 18 to 35 min^−1^ [38]. The normal heart rate range in young healthy adults is, depending on the fitness level, 40–100 bpm, and the respiration rate is between 12 and 18 min^−1^ with minor changes up to 45–60 years. Simply put, from a physiological point of view, these large differences between toddlers and adults can be explained by the metabolism of the healthy human body in terms of oxygen transport and consumption in relation to the size of the heart (more specifically stroke volume) and body surface area. Importantly, the faster the heart rate (HR), the shorter the time interval between two heartbeats for variability to occur (RRI (ms) = 60,000/HR (bpm)). The opposite pertains when the heart rate is low, which is called cycle length dependency. The immediate consequence for a study population comprising toddlers is that they have “less time for variability”. However, it is important to note that this is a nonlinear effect as can be seen in the formula for RRI: For instance, an increase of the heart rate from 60 to 62 bpm results from a shortening of the RRI by 32.26 ms; an increase of the HR from 100 to 102 bpm results from a shortening of the RRI by 11.76 ms. However, it should also be noted that the respiration frequency, too, is higher in toddlers than in adults, which results in shorter respiration cycles as well, and that the timings are perfectly coordinated under physiological conditions, whereas wrong timings can result in pathological conditions such as Cheyne–Stokes respiration.

In our healthy study populations, toddlers and adults showed the expected heart rates and respiration rates, which are in line with their age ranges. However, it is precisely this fact that causes problems in the frequency analysis. During sleep, the respiration rate in toddlers may be 18 min^−1^ or even lower, but it may be higher than 24 min^−1^ as well. That is, automatic segmental analysis of a 24 h ECG recording with a narrowly restricted frequency range would most probably fail or at least provide different information compared to SD1 (or RMSSD) [39]. Furthermore, this may even hold for pre-selected segments based on strict signal preprocessing and healthy adults, where information content may be lost when the HF band according to the recommended definition is used without further consideration.

### 4.2. Data Preprocessing

State-of-the-art ECG recordings go largely unnoticed shortly after placement of the equipment, which is of great advantage, particularly in small children. However, this lack of awareness can affect the signal quality, which thus must be expected to be lower in longer-term real-life recordings than under laboratory conditions. For this reason, the artifact detection and correction are crucial steps in data preprocessing, especially when it comes to ECG recordings over several hours [40,41].

In the present study, tried and tested, very strict artifact handling was employed [42,43,44]. Among other irregularities, exercising in adults or frequent changes in activity levels and body positions, as are particularly prevalent in toddlers, produce fast and significant heart rate changes. These may cause differences in information content between time-domain and frequency-domain variables. This can easily be figured when thinking of the quick physiological response of the heart rate to control the mean blood pressure (e.g., in response to changes of body positions), and its different impact on SDNN (which includes removal of the mean of all data points in the series) and the power spectral density (PSD), for which the signal is detrended before its calculation, i.e., a linear trend is subtracted from each data point. As a result, the frequency-based estimates of HRV are more susceptible to disturbances caused by sudden changes of body position and similar events than the time-based estimates (SDNN, RMSSD). To keep this source of error under control, we excluded all segments where the data trend reached a narrow predefined limit and additionally checked if the remaining segments fulfilled the Parseval’s theorem in order to ensure that the information content of the total PSD using Burg’s method is identical to the overall variance (SDNN^2^). However, while this was a vital step to ensure correct conclusions in the present study, in typical applications of the HRV, this criterion is a very strict one. Yet, it is imperative that any use of frequency-domain HRV includes a careful inspection of the course of the R-R intervals before the scores are calculated in order to make sure that the interpretation of the resulting band power values is of integrity.

Furthermore, it is important to be aware that the resampling method influences the result. Depending on the method of interpolation, additional spectral components may be brought in, which are not based on physiological processes but on mathematical artifacts. In former times, the simple but very efficient method introduced by Berger and co-workers [45] was used. However, according to more recent advances, piecewise cubic spline interpolation is the standard method. This also holds when it comes to the replacement of artifacts in the data series [41,46]. In addition, power spectral density estimates using the parametric autoregressive method depend on the model order. Following, for instance, the observations of Boardman and co-workers [47] as well as according to our own experience, we recommend Burg’s method with a model order of 24 [48]. Taken together with careful data preprocessing, much can be done to minimize interpretation errors that may occur for mathematical reasons alone.

A further important issue, specifically in the context of the adjustment of the HF band to the respiration frequency, is the correct detection of the respiration rate. Small and light devices such as the eMotion Faros 180^®^, which was used in the present study, have great advantages particularly for ECG recordings in small children, and 3D-acceleration sensors may be an additional asset. However, the light devices typically do not include a simple impedance measurement via the ECG electrodes for additional recording of the respiration, while, for instance, the larger CardioMem^®^ CM 4000 does (GE Healthcare, Freiburg, Germany). If the device does not provide a separate signal of respiration, it can be extracted offline from the ECG signal. For this purpose, the concept of the single-lead ECG-derived respiration (EDR) can be used, which was introduced by Moody and improved by others [49]. Using the EDR concept, we are extracting the respiration from the ECG signal offline in Matlab by means of a development of our research group, which in in the first step is based on the QRS complex in terms of pattern, slope, and amplitude. The recognition of possible respiration patterns is implemented after additional normalization of the band-pass filtered signal, where the limits are based on the physiological limits according to the participants’ age [38]. The algorithm was optimized during the last years with the aid of synchronized measurements of 3-lead ECG and respiration recording via the respiration belt, nasal thermistor, or thoracic impedance [42,50]. It is important to note that the respiration detection from ECG signals is prone to artifacts, especially in recordings made in the more turbulent daytime. Therefore, it should always be mentioned as a limitation. To be on the safe side as regards artifacts in the detection of respiration, we used the median of the detected respiration rate within each segment (rather than the arithmetic mean) for the adjustment of the HF band.

### 4.3. Accordance between Short-Term Variability Captured by the Specific HF Bands and SD1

For the discussion of the accordance between short-term variability variables in the frequency domain and SD1, it is needed to first discuss the properties of these variables after signal preprocessing.

#### 4.3.1. Filter Properties of SD1

Berntson and co-workers [23] analyzed the filter properties of RMSSD and concluded that the findings raise caveats in the applications and interpretation of the RMSSD statistic. From the mathematical connection between SD1 and RMSSD, it follows that these filter characteristics have to be taken into account for SD1 as well. We tackled this issue using another more empirically-driven approach from a mathematical point of view, which was based on long-term recordings of the ECG including adults as well as young children.

The notion that RMSSD (and SD1) are for the most part respiration-induced (but not attributed to some mathematical artifact such as cardiac aliasing) is corroborated by the fact that the crucial mathematical assumptions are fulfilled. It is common knowledge that a simple discrete differentiation, on which these variables are based, have high-pass characteristics. However, unlike most technical signals, the by nature time-variant signal complicates the description of the filter properties and its cut-off frequency, respectively. However, in physiological (i.e., not pathological) conditions, the cardiac frequency is more than twice the respiratory frequency, which is also evident from the present data. That is, from a technical point of view, the Nyquist criterion is fulfilled (see also Hildebrand [51]).

By simple consideration of the formula “RMSSD equals the square root of the mean squared differences of successive R-R intervals” and the cycle length dependency, the following can be deduced: In a specific time segment in which the heart rate is faster, “more” short R-R intervals with “less time to vary” and “less” longer RRI with “more time to vary” are present compared to segments with a slower heart rate. By squaring the differences of successive R-R intervals, the impact of the rare longer RRI in such a time segment is accentuated, which adds to the uncertainty of the filter property of RMSSD or SD1. On the other hand, the differences of successive R-R intervals (a) can be further processed in the same manner as the absolute R-R intervals (important: albeit without detrending) and at the same time (b) the use of the differences of successive R-R intervals focus the statistic upon higher-frequency portions of the variability, which are attributed to influences of the nervus vagus. To conclude, even if certain mathematical imprecision is caused by the resampling (more so, when longer R-R intervals are present), this effect is vanishingly small. From this, it follows that Figure 3 adequately represents the frequencies that go into RMSSD and SD1 and, therefore, the use of SD1 in the accordance analysis (Section 3.1) is appropriate.

#### 4.3.2. Differences in Levels of Absolute Band Power in HF1 to HF6

It can be seen from Figure 1 that in toddlers during sleeping hours, the relative band power of short-term variability is adequately captured by the proposed frequency band of 0.15–0.80 Hz [30,31]. This was confirmed by the analysis of the absolute values (Section 3.3., Table 3), which showed low gain brought by adding the band power of 0.80–1.04 Hz. This is the expected finding considering the respiratory rate. Nevertheless, it is justifiable to set the upper frequency limit to 1.04 Hz, and the higher limit may provide additional information in the daytime. By contrast, our results indicate that the proposed bottom frequency limit of 0.24 Hz for children [35,36,37] may result in a loss of information content in toddlers, particularly in recordings in the daytime, although its physiological origin is still under debate [52]. Rather unexpectedly, the suggested adjustment of the HF band to the respiratory frequency [21,53] might result in information loss as well, even if dynamic adjustment within the individual is implemented, as can be seen in Table 2. This may corroborate the supposition of Porges [54] that the selection of a frequency band adequately representing the vagal proportion of the HRV may also depend on parameters of development and context (e.g., physical activity). However, in the context of our study, this means that the information lost by usage of the dynamically adjusted variables very likely is not driven by the respiration. Compared to the frequency-based variables, RMSSD [54] and therefore also SD1 were found to be less affected by the absolute levels of respiratory frequency and tidal volume [55]. For this reason, Laborde and co-workers [21] proposed to always validate the HRV frequency analysis, e.g., with RMSSD. Berntson and co-workers [23], on the other hand, recommended being cautious in the applications and interpretation of RMSSD, because RMSSD might be contaminated by sympathicus-driven (lower-frequency) variability. 

At least in adults, notable portions of variability in the frequency range < 0.15 are indeed present in SD1/RMSSD (see Figure 3). However, these oscillations are not necessarily of sympathetic nature [56], which is a notion that has been widely accepted in the meantime.

#### 4.3.3. Accordance between Specific HF Bands and SD1 and Derivation of Specific Recommendations

A majority of studies are done under well-designed and controlled laboratory conditions, and we are positive that the common analysis standards are adequate under these circumstances. However, our study demonstrates that the situation is different in real-life conditions and that standards should be adapted when children are examined, because in these cases, interpretation errors may otherwise occur. We found that a dynamic adjustment of the HF band based on the current individual respiration rate does not necessarily result in a higher accordance of the HF power with SD1 in that individual. At first glance, this may seem unexpected and contradictory to the recommendation to adjust the HF band for respiration frequency [21,57,58]. However, the imperfect correlation gives a hint that in certain conditions, these variables, originating from different mathematical spaces, do not share 100% of their information content. Moreover, the lower correlation of HF5 (bandwidth 0.25 Hz) than HF6 (bandwidth 0.65 Hz) with SD1 demonstrates that the restriction to a defined band width is a limitation, with an additional implicit restriction brought about by the high-pass property of SD1.

Taken together, the HRV analysis of ECG data acquired during sleep is less error-prone compared to long-term data recorded in the daytime, irrespective of whether the HF band is dynamically adapted to the individual respiratory frequency or a broad fixed HF band is used. As regards the latter, it is important to take into account that the respiratory frequency of toddlers can be less than 18 min^−1^ (see Figure 1) and, therefore, the lower cut-off frequency should be set to 0.15 Hz, just as in adults. While in children, an extension of the upper frequency limit to 0.80 Hz or more has been recommended before [33,34,35,36,37], the accordance with SD1 was improved by moving the higher cut-off frequency from 0.40 to 0.80 Hz also in adults (see Table 1 and Figure 2 and Figure 3 combined). The physiological origin of these fast oscillations in adults need to be investigated, because the average respiratory frequency is not that high in healthy adults. A probable cause is the (common and benign) irregularities in the respiration, which introduce fast frequency components into the signal. Compared to adults, toddlers showed a remarkable large share of variability in these high spectral components in the range of 0.40–0.80 Hz, which is to be expected because of their overall higher respiratory frequency. In conclusion, for recordings during sleeping hours, the use of a broad HF band ranging from 0.15 to 0.80 Hz seems appropriate in adults as well as in toddlers. RMSSD, which is easier to calculate and in healthy individuals covers the same information as the frequency-domain based band power in the recommended range, is equally appropriate.

The matter is more complex in the daytime. In line with Berntson et al. [23], we observed that RMSSD (SD1) showed clear high-pass filter properties, which also let lower-frequency fluctuations (<0.15 Hz) through. The occurrence of lower frequencies was more pronounced in adults than in toddlers (Figure 3). As the definitions of the HF bands do not include these lower-frequency components, they reduce the correlations between RMSSD and the HF band power to <1.0. Moreover, as the variability in frequencies lower than 0.15 Hz increases in more active periods, the size of the correlations obtained in the daytime unsurprisingly do not reach that of the coefficients during sleep. However, for recordings during the day, it is difficult to give clear recommendations for the most appropriate HF range in adults and toddlers, because it depends on the individual’s activity levels during the recording day, which can vary widely within as well as between individuals. The best compromise in adults is to use a broad frequency band, ranging from 0.15 to 0.8 Hz: in this broad HF band, all essential components are included, no matter whether the person was resting or even sleeping or very active and in which proportions these states occurred during the recording day. When analyzing data of young children recorded during the day, the HF band should be moved upwards compared to the frequency band for sleeping hours, allowing for children’s higher respiratory frequency in the daytime. Here, the best choice is to use a broad HF band ranging from 0.24 to 1.04 Hz, if there is a desire to use frequency-based HRV variables at all. Again, RMSSD implicitly meets the requirements due to its filter properties, which automatically take the R-R intervals into account. Therefore, our recommendation is to stay with the time-domain variables unless a highly refined analysis is needed, which may require more mathematical and physiological expertise.

## 5. Conclusions

Recent developments in noninvasive ECG monitoring with small, wearable sensors open the opportunity to record high-quality ECG over many hours in an easy and non-burdening way. However, the present study demonstrated that even well-established frequency-domain variables of heart rate variability may fail if basic mathematical and physiological principles are not considered. The major conclusion derived from the present findings is that for most applications that implicate long-term monitoring across varying circumstances and activity levels in healthy individuals, the time-domain variables are adequate to gain an impression of an individual’s heart rate variability and, thus, the dynamic adaptation of an organism’s behavior in response to the ever-changing demands of daily life. The interpretation of frequency-domain variables requires more consideration of physiological and mathematical principles and a careful selection of frequency bands which, depending on the recording conditions and study populations, may go well beyond common recommendations. Nevertheless, if users prefer to use frequency-domain variables, the selection of frequency bands clearly must undergo informed adaptation, particularly if data are recorded in real-life conditions compared to measurements in the laboratory, as well as when children are examined instead of adults. Otherwise, interpretation errors are likely. Matters are somewhat less complex in recordings during sleeping hours, where a broad high-frequency band ranging from 0.15 to 0.80 Hz may be useful in adults and children. During the day, it depends on the activity level of the individual during the recording, which frequency range most adequately captures the relevant high-frequency heart rate variability. An acceptable compromise for daytime recordings in adults may be to use a broad frequency band ranging from 0.15 to 0.80 Hz, which includes all essential components of high-frequency variability. In young children, the higher respiratory frequency calls for moving up the range of the high-frequency band to 0.24 to 1.04 Hz. However, unless a highly refined analysis of heart rate variability is in the focus of interest, on the basis of the elaborate analysis in this paper, the use of RMSSD is to be recommended. The time-domain variable RMSSD is adequate and most unproblematic also in longer-term recordings and more real-life conditions implicating varying activity levels, which can be adequately interpreted in adults as well as in children.

## Figures and Tables

**Table 1:** Mean correlation coefficients (Fisher’s Z’) between SD 1 and absolute band power in the six HF bands ^1^ (mean ± SD).

	Analysis 1: Sleep vs. Daytime ^2^		Analysis 2: Low vs. High Activity ^3^	
	Sleep	Daytime	Low Activity	High Activity
*Z’_(ln(SD1)__∗__ln(HF1))_*				
toddlers	2.06 ± 0.48	1.59 ± 0.32	1.51 ± 0.37	1.23 ± 0.21
adults	2.15 ± 0.38	1.97 ± 0.28	1.84 ± 0.38	1.61 ± 0.35
*Z’_(ln(SD1)__∗__ln(HF2))_*				
toddlers	2.47 ± 0.39	2.05 ± 0.36	2.04 ± 0.41	1.61 ± 0.29
adults	2.28 ± 0.38	2.23 ± 0.29	2.05 ± 0.36	1.92 ± 0.33
*Z’_(ln(SD1)__∗__ln(HF3))_*				
toddlers	2.30 ± 0.45	2.26 ± 0.35	2.16 ± 0.40	1.92 ± 0.26
adults	1.40 ± 0.56	2.27 ± 0.35	1.92 ± 0.36	2.05 ± 0.35
*Z’_(ln(SD1)__∗__ln(HF4))_*				
toddlers	2.51 ± 0.38	2.13 ± 0.36	2.11 ± 0.40	1.71 ± 0.28
adults	2.28 ± 0.38	2.27 ± 0.30	2.06 ± 0.37	1.99 ± 0.31
*Z’_(ln(SD1)__∗__ln(HF5))_*				
toddlers	1.99 ± 0.48	1.63 ± 0.36	1.51 ± 0.43	1.29 ± 0.23
adults	2.13 ± 0.37	1.90 ± 0.30	1.63 ± 0.33	1.05 ± 0.31
*Z’_(ln(SD1)__∗__ln(HF6))_*				
toddlers	2.35 ± 0.36	2.14 ± 0.35	2.05 ± 0.41	1.78 ± 0.26
adults	2.26 ± 0.37	2.10 ± 0.32	1.78 ± 0.33	1.76 ± 0.31

^1^ Correlations were computed for each participant and Fisher’s Z’ was transformed to enable inferential statistical analysis (see Section 2.3.4). ^2^ Significant three-way interaction frequency band × time of day × age group (*F*(5,465) = 41.7, *p* < 0.001). ^3^ Significant three-way interaction frequency band × activity level × age group (*F*(5,465) = 15.2, *p* < 0.001).

**Table 2:** Basic cardiac and respiration characteristics in toddlers and adults (mean ± SD).

	Analysis 1: Sleep vs. Daytime ^1^		Analysis 2: Low vs. High Activity ^2^	
	Sleep	Daytime	Low Activity	High Activity
*Heart rate (bpm)*				
toddlers	96.0 ± 9.3	122.0 ± 8.4	115.4 ± 8.7	131.2 ± 8.9
adults	61.4 ± 7.1	92.2 ± 11.7	81.9 ± 10.0	107.3 ± 16.6
*SDNN (ms)*				
toddlers	60.6 ± 37.5	36.0 ± 13.5	39.3 ± 16.4	31.5 ± 10.6
adults	77.9 ± 23.8	58.6 ± 16.7	69.1 ± 18.7	43.2 ± 19.2
*ln(SD1) (ms)*				
toddlers	3.35 ± 0.76	2.64 ± 0.48	2.82 ± 0.53	2.38 ± 0.43
adults	3.72 ± 0.51	2.88 ± 0.46	3.22 ± 0.43	2.36 ± 0.61
*ln(SD2) (ms)*				
toddlers	4.09 ± 0.52	3.76 ± 0.30	3.83 ± 0.33	3.68 ± 0.29
adults	4.40 ± 0.32	4.23 ± 0.27	4.45 ± 0.24	3.92 ± 0.43
*Respiration rate (min^−1^)*				
toddlers	21.7 ± 1.9	27.1 ± 1.7	26.5 ± 1.8	27.9 ± 1.7
adults	15.6 ± 2.0	20.7 ± 1.1	19.4 ± 1.1	22.7 ± 1.7

^1^ Significant differences between toddlers and adults (*p* ≤ 0.005) in all variables. Significant two-way interactions time of day × age group in the analyses of heart rate (*F*(1,93) = 7.7, *p* = 0.007) and long-term variability (SD2; *F*(1,93) = 6.6, *p* = 0.012). ^2^ Significant differences between toddlers and adults (*p* < 0.001) in all variables except SD1. Significant two-way interactions activity level × age group (*p* < 0.001) in all variables.

**Table 3:** Absolute band power in the predefined frequency bands (mean ± SD).

	Analysis 1: Sleep vs. Daytime ^1^		Analysis 2: Low vs. High Activity ^2^	
	Sleep	Daytime	Low Activity	High Activity
*ln(HF1) (ms^2^)*				
toddlers	6.43 ± 1.50	4.82 ± 0.93	5.21 ± 1.02	4.28 ± 0.88
adults	6.90 ± 1.02	5.35 ± 0.80	6.02 ± 0.73	4.34 ± 1.16
*ln(HF2) (ms^2^)*				
toddlers	6.63 ± 1.44	5.23 ± 0.92	5.61 ± 1.00	4.70 ± 0.87
adults	6.99 ± 1.00	5.52 ± 0.79	6.15 ± 0.74	4.58 ± 1.12
*ln(HF3) (ms^2^)*				
toddlers	6.45 ± 1.43	4.89 ± 0.96	5.29 ± 1.05	4.33 ± 0.89
adults	6.15 ± 0.93	4.86 ± 0.88	5.47 ± 0.84	3.94 ± 1.14
*ln(HF4) (ms^2^)*				
toddlers	6.64 ± 1.43	5.28 ± 0.91	5.65 ± 1.00	4.76 ± 0.86
adults	6.99 ± 1.00	5.54 ± 0.79	6.16 ± 0.74	4.81 ± 1.10
*ln(HF5) (ms^2^)*				
toddlers	6.39 ± 1.48	4.38 ± 1.05	4.88 ± 1.14	3.69 ± 1.02
adults	6.90 ± 1.02	5.05 ± 0.89	5.85 ± 0.80	3.86 ± 1.31
*ln(HF6) (ms^2^)*				
toddlers	6.54 ± 1.47	4.87 ± 0.97	5.29 ± 1.07	4.30 ± 0.91
adults	6.98 ± 1.00	5.26 ± 0.86	5.99 ± 0.79	4.17 ± 1.22

^1^ Significant three-way interaction frequency band × time of day × age group (*F*(5,465) = 15.5, *p* < 0.001). ^2^ Significant three-way interaction frequency band × time of day × age group (*F*(5,465) = 22.0, *p* < 0.001).
